# EDITORIAL: Molecular Pharmacology of the GABA_A_ Receptor

**DOI:** 10.2174/157015910790909539

**Published:** 2010-03

**Authors:** GUSTAV AKK

The GABA_A_ receptor is a member of the Cys-loop family of transmitter-gated ion channels. GABA_A_ receptors respond to synaptically-released or ambient transmitter with a conformational change, resulting in the opening of the gate that allows movement of small anions through the channel. Under most circumstances, GABA_A_ receptor-mediated signaling leads to hyperpolarization in the mature brain. The normal functioning of the receptor is critical to cellular function, while errors in GABA_A_ receptor activity can systemically manifest as seizures, anxiety disorders and others. The pharmacology of the GABA_A_ receptor is remarkably rich. The receptor is a target for benzodiazepines, steroids, barbiturates, and a number of other classes of compounds, some of which are used clinically as anesthetics, anxiolytics, or anticonvulsants. The actions of the drugs are in many cases dependent on the subunit composition of the GABA_A_ receptor. Thus, understanding how the existing therapeutics act can pave way to new drugs with improved specificity and reduced side effects. This Hot Topic Issue presents an overview of molecular pharmacology of the GABA_A_ receptor focusing on three classes of positive modulators: general anesthetics, benzodiazepines, and neuroactive steroids.

**Paul Garcia, Scott Kolesky and Andrew Jenkins** provide an introduction to GABA_A_  receptors, and discuss the actions of major classes of volatile and intravenous anesthetics. The manuscript addresses the relationship between subunit composition of the receptor and modulation, and summarizes the data from work on mutated receptors on the location of the anesthetic binding cavity. **Matt Bianchi** presents a summary of the actions of benzodiazepines on the GABA_A_  receptor. The major effect of benzodiazepines is to enhance the affinity of the receptor to the transmitter. The manuscript is aimed at comparing and contrasting the ability of benzodiazepines to modulate currents under conditions resembling synaptic conditions and tonic exposure to GABA. Finally, **Gustav Akk** and colleagues discuss the current knowledge about kinetic and structural features of GABA_A_  receptor modulation by potentiating neuroactive steroids. This class of steroids acts by increasing channel open probability through a set of modifications in the channel open and closed time distributions. The review critically evaluates available experimental data discussing the possibility of a single vs. multiple interaction sites mediating the effects of potentiating steroids.

This Hot Topics Issue provides an overview of the rapidly developing field of molecular pharmacology of the GABA_A_  receptor. The manuscripts focus on several of the numerous positive modulators of the receptor summarizing the present state of knowledge and introducing new evidence and novel viewpoints about the kinetic and structural aspects of modulation. It is hoped that the presented material will provide an impetus for further research in this area.

